# Outcomes in Heart Failure With Improved Ejection Fraction Following Implantable Cardioverter-Defibrillator Placement for Primary Prevention

**DOI:** 10.7759/cureus.85360

**Published:** 2025-06-04

**Authors:** Michael N Zarrella, Carolina Borz-Baba, Dorothy Wakefield, Kolu Wynne, Kevin Kett

**Affiliations:** 1 Internal Medicine, St. Mary's Hospital, Waterbury, USA; 2 Statistics, St. Francis Hospital, Hartford, USA

**Keywords:** brain natriuretic peptide (bnp), heart failure with improved ejection fraction (hfimpef), hospitalization outcomes, implantable cardioverter-defibrillator (icd), mortality

## Abstract

Background

Limited data exist on outcomes in heart failure with improved ejection fraction (HFimpEF) following implantation of cardiac devices, such as implantable cardioverter-defibrillator (ICD) or cardiac resynchronization therapy (CRT). In this contemporary analysis, we utilize the most current American Heart Association (AHA)/American College of Cardiology (ACC) 2022 definition of HFimpEF to evaluate hospitalization and mortality in this population.

Methods

This retrospective study analyzed patients who received ICD or CRT for primary prevention at a non-tertiary hospital between 2019 and 2022. Data were extracted from the Device Implant Registry, assessing demographics, clinical parameters, echocardiography, and device type. Improvement in ejection fraction (EF) was assessed using a follow-up echocardiogram performed at least six months after device implantation. The outcomes measured included the one-year and overall (3.6 years) rates of hospitalization and mortality from the date of ICD/CRT insertion to October 1, 2024.

Results

Our study comprised 54 patients with a repeat echocardiogram after at least six months post-ICD/CRT placement to assess left ventricular ejection fraction (EF). Those with HFimpEF had fewer one-year admissions (22.2%) compared to those with persistent heart failure with reduced EF (HFrEF) (48.2%) (p < 0.05). Overall hospitalization rates were also significantly lower (59.3%) in HFimpEF versus 85.19% in HFrEF (p < 0.05). Heart failure accounted for 50% of the cardiac-related hospitalizations. Mortality rates were reduced (14.8%) in the HFimpEF group compared with 29.63% in HFrEF, but there was no significant statistical difference between the groups. A lower brain natriuretic peptide (BNP) is predictive of both improved EF (p < 0.01) and decreased mortality (p < 0.01).

Conclusions

Patients with myocardial recovery post-ICD/CRT have better prognoses than those with persistent HFrEF. Uncontrolled heart failure remains the predominant factor contributing to hospitalization after device implantation among our cohort. Implementing an outpatient standardized clinical interval summary that incorporates scheduled BNP assessment may represent a cost-effective strategy to enhance the care of patients with HFimpEF in non-tertiary healthcare.

## Introduction

Heart failure with improved ejection fraction (HFimpEF) has emerged as a new clinical entity evolving from the long-term progression of heart failure with reduced ejection fraction (HFrEF) (ejection fraction (EF) < 40%) in the era of goal-directed medical therapy (GDMT) and device interventions. According to the American Heart Association (AHA)/American College of Cardiology (ACC) 2022 guidelines, HFimpEF is defined as an initial EF below 40% followed by a subsequent improvement in EF to greater than 40% [1]. For over a decade, considerable interest has been devoted to identifying predictors of left ventricular ejection fraction (LVEF) recovery and evaluating outcomes in patients with HFimpEF, including those who received implantable cardioverter-defibrillator (ICD) or cardiac resynchronization therapy (CRT) implantation. However, the majority of studies have employed varying criteria to describe HFimpEF [2-22]. The existing literature on outcomes following myocardial recovery with device therapy remains controversial. The inconsistencies observed across the studies are not primarily due to the type of device alone, but stem from a combination of factors, including variability in follow-up duration, differences in the use or de-escalation of GDMT, and heterogeneity within the patient population. Our analysis applies the latest definition of HFimpEF to evaluate hospitalization and mortality outcomes in patients with different implanted cardiac devices.

## Materials and methods

Study design

This IRB-approved retrospective study was conducted at a non-tertiary hospital, examining patients with HFrEF ≤ 35% who underwent implantable cardioverter-defibrillator (ICD) or cardiac resynchronization therapy (CRT) implantation for primary prevention between January 1, 2019, and December 31, 2022. Patient data were obtained from the Device Implant Registry, capturing demographics, comorbidities, echocardiographic parameters, medication adherence to GDMT, and device type. Additional clinical details were extracted through chart review.

Left ventricular ejection fraction (EF) was measured using 2D transthoracic echocardiography, recorded within three months prior to implantation, and reassessed again six to 36 months post-procedure. Per AHA/ACC guidelines, HFimpEF was defined as a left ventricular ejection fraction (LVEF) that improved to greater than 40%. The most recent echocardiogram was used to determine each patient's classification as either having persistently reduced EF (HFrEF) or improved EF (HFimpEF). Among patients with multiple echocardiograms during the study period, no significant fluctuations or reversals in EF trajectory were observed, supporting the stability of their classification. Patients received either an ICD (dual chamber: 58%, single chamber: 22%) or CRT-D (20% of total devices) and were on guideline-directed medical therapy (GDMT) for at least three months prior to implantation. GDMT was continued post-device implantation regardless of EF recovery. Those without a post-implant echocardiogram were excluded. Changes in GDMT following ICD implantation could not be assessed due to the long-term nature of the study and logistical limitations of the EPIC system. However, all patients in this study were on maximally tolerated GDMT at the time of device implantation and advised to maintain therapy indefinitely.

Patient characteristics

The study included adult patients between the ages of 18 and 99 years and evaluated several clinical variables. Demographic information included age, gender, and ethnicity. Clinical comorbidities included the following: ischemic cardiomyopathy, prior coronary artery bypass grafting (CABG), percutaneous coronary intervention (PCI), atrial fibrillation, hypertension, dyslipidemia, type 2 diabetes mellitus (both insulin-dependent and non-dependent), end-stage renal disease on hemodialysis (ESRD on HD), and chronic lung disease (asthma, chronic obstructive lung disease, obstructive sleep apnea). Echocardiographic data and laboratory markers included left ventricular end diastolic diameter (LVEDD), left ventricular end systolic diameter (LVESD), and B-type natriuretic peptide (BNP).

BNP levels were assessed over a 3.6-year period following ICD implantation. In evaluating each patient group (HFimpEF or HFrEF), the highest and lowest recorded values were collected, and a median was calculated to characterize the individual’s BNP profile. Patient-level medians were then used to generate group-level analysis (median and interquartile range) for the HFimpEF and HFrEF to explore their association with mortality.

Outcomes

Primary outcomes compared HFimpEF versus HFrEF regarding rates of hospitalization and mortality. Hospitalization rates were compared at one year and at 3.6 years (overall). Mortality rates were assessed over a 3.6-year follow-up period.

Statistical analysis

Descriptive statistics were applied to clinical and demographic variables. Group comparisons used chi-square tests for categorical data and Fisher’s exact tests for small cell sizes (<5). Age differences were analyzed using t-tests, with statistical significance set at p < 0.05 (SAS 9.4, SAS Institute, Cary, USA).

## Results

Patient characteristics****

The cohort study included 54 patients, with a male predominance. As outlined in Table 1, more than half of the patients had a documented history of ischemic cardiomyopathy, hypertension, dyslipidemia, and marked functional impairment (New York Heart Association (NYHA) Class III). The prevalence of atrial fibrillation and left bundle branch block in our cohort was below 25%. The mean LVEDD was at the upper limit of normal (≤5.6 cm), but the mean LVESD was larger than normal (≤4.0 cm). Additionally, the median BNP was 806.5 pg/mL. Among the predictive factors collected for our study population, as seen in Table 2, the sole parameter that demonstrated statistical significance in predicting myocardial recovery was a lower BNP.

**Table 1:** Patient Characteristics CABG: coronary artery bypass grafting, PCI: percutaneous coronary intervention, BNP: brain natriuretic peptide, NYHA: New York Heart Association.

Patient Characteristics	All Patients (n = 54)
Age, years, mean (SD)	65.9 (±11.4)
Male gender, n (%)	39 (72%)
Ischemic cardiomyopathy, n (%)	38 (70%)
Prior CABG, n (%)	6 (11%)
Prior PCI, n (%)	27 (50%)
Atrial fibrillation, n (%)	12 (22%)
Hypertension, n (%)	42 (77%)
Dyslipidemia, n (%)	48 (89%)
Diabetes mellitus, n (%)	25 (46%)
Chronic lung disease, n (%)	11 (20%)
End-stage renal disease on hemodialysis, n (%)	2 (4%)
Left bundle branch block, n (%)	12 (22%)
NYHA Class II, n (%)	20 (37%)
NYHA Class III, n (%)	28 (52%)
NYHA Class IV, n (%)	6 (11%)
Left ventricular end-diastolic diameter in cm (SD)	5.5 (±0.77)
Left ventricular end-systolic diameter in cm (SD)	4.7 (±0.85)
BNP, median (25th-75th Pctl)	806.5 (154-2,320)
QRS, median (IQR)	114 (92-154)

**Table 2:** Characteristics of Patients With HFimpEF Versus HFrEF HFimpEF: heart failure with improved ejection fraction, HFrEF: heart failure with reduced ejection fraction, CABG: coronary artery bypass grafting, PCI: percutaneous coronary intervention, ESRD: end-stage renal disease, LBBB: left bundle branch block, LVEDD: left ventricular end diastolic diameter, LVESD:  left ventricular end systolic diameter, BNP: B-type natriuretic peptide.

	HFimpEF	HFrEF	P-value
Number (n = 54)	27	27	
Age in years ± SD	63.9 ± 10.8	67.9 ± 11.8	0.20
Male gender	18 (66.7%)	21 (77.8%)	0.36
Ischemic cardiomyopathy	18 (66.7%)	20 (74.1%)	0.55
Prior CABG	5 (18.5%)	1 (3.7%)	0.08
Prior PCI	12 (44.4%)	15 (55.6%)	0.49
Atrial fibrillation	7 (25.9%)	5 (18.5%)	0.51
Hypertension	20 (74.1%)	22 (81.4%)	0.51
Dyslipidemia	22 (81.4%)	26 (96.3%)	0.08
Diabetes mellitus	14 (58.3%)	11 (40.1%)	0.41
Chronic lung disease	5 (18.5%)	6 (22.2%)	0.74
ESRD on hemodialysis	1 (3.7%)	1 (3.7%)	1.00
LBBB	6 (22%)	6 (22%)	1.00
LVEDD mean in mm (SD)	5.49 (±0.72)	5.65 (±0.83)	0.46
LVESD mean in mm (SD)	4.63 (±0.9)	4.88 (±0.77)	0.27
BNP, median (IQR)	332 (64,1304)	1751 (525,4700)	0.01

Hospitalization and mortality

Hospitalizations at one year and over a median follow-up of 3.6 years were significantly lower among patients who exhibited EF recovery (Table 3). Notably, heart failure-related hospitalizations and overall mortality were less prevalent in HFimpEF than in HFrEF, although it did not reach statistical significance. Patients that died were older (p < 0.05), more likely to have chronic lung disease (p < 0.05), ESRD on HD (p < 0.05), and a higher BNP (p < 0.05) (Table 4).

**Table 3:** Hospitalization and Mortality HFimpEF: heart failure with improved ejection fraction, HFrEF: heart failure with reduced ejection fraction.

	HFimpEF	HFrEF	P-value
1-year hospitalization, n (%)	6 (22.2%)	13 (48.2%)	0.04
Overall hospitalization, n (%)	16 (59.3%)	23 (85.2%)	0.03
Overall mortality, n (%)	4 (14.8%)	8 (29.6%)	0.19

**Table 4:** Predictors of Mortality ESRD: end-stage renal disease on hemodialysis, BNP: B-type natriuretic peptide.

	Survived	Deceased	P-value
Age, year, mean (SD)	64 (±10.5)	72 (±12.4)	0.03
Chronic lung disease	5 (11.9%)	6 (50%)	0.01
ESRD on HD	0	2 (16.7%)	0.04
BNP, median (IQR)	428 (72,888)	2,945 (2,213, 4,700)	0.03

## Discussion

Prevalence and incidence of HFimpEF post-device implant

The prevalence and incidence of EF improvement following device implantation vary across studies. In 2018, the CARDIOCHUS-CHOP study reported a 28% incidence of LVEF recovery, suggesting a potential association between EF improvement and the absence of defibrillators [7]. However, this finding was challenged by the largest study focused exclusively on patients with an ICD for primary prevention, which identified that partial (EF > 35% and ≤ 50%) and complete (≥ 50%) EF recovery was observed in 24.8% of cases [21]. Over a median follow-up of 4.9 years, the incidence of left ventricular (LV) myocardial recovery reached 40% [21], although it declined to 28% over a decade in non-CRT super-responders [23]. These findings indicate that myocardial recovery may be transient or fluctuate around the 40% threshold [24].

In our cohort, 50% of patients experienced EF improvement over 3.6 years, a proportion exceeding that reported in previous studies. This discrepancy is likely attributable to the inclusion of patients with CRT (16.7%), which independently facilitates myocardial remodeling and recovery [19,25].

Demographic and clinical predictors of HFimpEF post-device implant

Demographic and clinical predictors of myocardial recovery in patients with device therapy are consistent with those observed in the non-device population, including younger age [21], female gender, de novo HF, non-ischemic cardiomyopathy, left bundle branch block (LBBB), and NYHA Class II [3,19,26,27]. Our cohort was predominantly composed of elderly male patients with advanced HF (NYHA Class III-IV), ischemic cardiomyopathy, and a lower prevalence of LBBB, preventing our ability to confirm these associations.

Certain comorbidities, such as hypertension, diabetes, and renal failure requiring dialysis, have been linked to persistently reduced EF [15]. However, multiple subsequent analyses did not establish a negative correlation between controlled comorbidities, including hypertension, hyperlipidemia, atrial fibrillation (AF), and diabetes mellitus (DM), and myocardial recovery [18,23,26]. Similarly, our study indicates that coexisting comorbidities do not preclude EF improvement following ICD implantation.

Echocardiographic predictors

A larger LVEDD and LVESD have been negatively correlated with myocardial recovery [3], although our study was unable to confirm this association due to sample size limitations. Additional emerging echocardiographic parameters such as mechanical dispersion (MD) are increasingly recognized for their prognostic value of arrhythmias, reinforcing the necessity of continued device therapy despite EF improvement [28-30]. Novel metrics, MD and relative wall thickness (RWT), offer practical benefits and can be seamlessly integrated into routine echocardiographic evaluations even in non-tertiary hospitals.

Biomarkers

While new biomarkers such as soluble suppression of tumorigenicity 2 (sST2) and Galectin-3 show promise in assessing cardiac remodeling, their limited availability in community settings constrains their clinical utility [3]. Conversely, widely accessible biomarkers like N-terminal pro-brain natriuretic peptide (NT-proBNP), BNP, and troponin remain valuable tools for assessing myocardial recovery. Lower BNP and troponin levels have been associated with EF improvement [3], although their role post-device implantation remains insufficiently explored. In our study, lower BNP levels correlated positively with EF improvement, highlighting the relevance of this biomarker in guiding treatment optimization, even in the absence of overt clinical deterioration. The results of this study indicate that routine BNP assessment every six to 12 months, regardless of clinical symptomatology, may prove valuable. They could supplement or even replace routine TTE as part of long-term monitoring.

Post-device therapy hospitalizations

Myocardial recovery has been consistently linked to better clinical outcomes, including a reduction in hospitalizations [2,3,5-8,10]. Device therapy plays a pivotal role in minimizing HF-related admissions, with ICDs and CRT outperforming medical therapy alone [17]. Data from the American College of Cardiology’s ICD registry found that HFimpEF patients had significantly fewer hospitalizations (12.2%) compared to those with persistently reduced EF (27.5%) [15]. Similarly, a German cohort reported a hospitalization rate of 9.5% in HFimpEF compared to 38% in those with persistent HFrEF [24].

Our findings align with these studies, demonstrating a statistically significant reduction in hospitalization at one year (p < 0.05) and over a 3.6-year follow-up (p < 0.05) among patients receiving device therapy. This improvement is likely due to better medication adherence, optimized chronic disease management, and the correction of reversible contributors to adverse remodeling, such as tachycardia and toxic exposures. Consistent with prior research, cardiac-related admissions (54%) were mainly due to HF exacerbations (43%)[15,24]. Notably, patients with multiple admissions (15%) were all hospitalized within the first year, with 67% experiencing at least one congestive heart failure (CHF)-related admission, and all succumbed by the end of the follow-up.

Equally important, the findings underscore the ongoing challenge of managing heart failure to prevent hospitalizations after device therapy. This includes continued GDMT despite EF recovery as recommended by AHA guidelines. Regular outpatient follow-up, which includes monitoring adherence, performing repeat imaging to detect early ischemic or valvular events, interrogating the device, and engaging in shared decision-making for advanced care planning, is essential.

Post-device therapy mortality

Mortality rates in HFimpEF patients reflect the combined effects of CRT on reverse remodeling and ICD therapy in preventing sudden cardiac death (SCD). Reported annual mortality ranges from 3% to 12%, with HF as the leading cause of death and SCD comprising a smaller proportion [17]. However, mortality comparisons between persistently low EF and improved EF patients vary, with reported rates ranging from 3.8% over 4.9 years [21] to 10% over 3.8 years [24] reaching 23% in some studies [15]. Certain analyses, including Madhavan et al. [23], found no significant mortality difference after five years between patients with EF >35% and those with EF ≤35% (p = 0.68), an observation consistent with the Sudden Cardiac Death in Heart Failure trial.

Two Key Factors Influence Post-device Mortality

Patient phenotype: In our cohort, 22% of patients died over 3.6 years, with a 50% relative risk reduction observed among those with EF improvement. Heart failure was the predominant cause of death. Patients who died were older (p < 0.05), had chronic respiratory conditions (p < 0.05), and were more likely to require dialysis (p < 0.05), observations that are grounds for further investigations. Elevated BNP levels retained strong prognostic significance (p < 0.01), reinforcing the importance of integrating biomarkers into risk stratification models to optimize resource allocation, particularly for elderly patients with mobility and cognitive limitations.

Unmodifiable contributors: Ventricular arrhythmias [14,17] and appropriate ICD shocks [21,23] significantly influence mortality risk. Although primary prevention ICD discharges remain infrequent [17], they can still impact outcomes. In our cohort, two patients with persistently low EF experienced ICD shocks for ventricular arrhythmias, both of whom subsequently died. The occurrence of shocks is a marker of amplified risk, emphasizing the importance of vigilant monitoring of patients who had received both appropriate and inappropriate shocks.

Despite improved survival in patients with myocardial recovery post-device therapy, overall mortality remains high due to advanced age, chronic comorbidities, HF progression, and persistent arrhythmic risk. Notably, as ICD therapy reduces arrhythmic mortality, HF-related deaths have become predominant in this population.

Risk stratification and follow-up care

A robust outpatient risk stratification model incorporating clinical factors (age, comorbidities, EF status, shocks) and biomarkers (e.g., BNP) is paramount for predicting hospitalization and mortality. Implementing BNP assessment every six months and prompting a clinical visit in response to rising levels is cost-effective and can be incorporated into clinical decision pathways, allowing early identification of potential decompensation and timely intervention to prevent hospitalization. It supports optimal resource utilization in heart failure clinics by facilitating targeted patient education, structured follow-up to evaluate advanced interventions such as a Left Ventricular Assist Device versus transplant referral, and coordinated care with primary care providers to ensure adequate management of comorbidities.

High-risk patients, especially the elderly with multiple comorbidities, benefit from advanced surveillance programs to identify clinical deterioration, early detection of ischemic or valvular events, device interrogations, and adherence monitoring are critical. A one- to three-month interval would be a practical approach to allow shared decision-making regarding prognosis and advanced care planning for patients with advanced HF and limited functional status.

Strengths and limitations

Strengths

This study is one of the first to analyze outcomes in HFimpEF patients post-ICD/CRT implantation using the 2022 definition of HFimpEF by the American Heart Association (AHA)/American College of Cardiology (ACC), which minimizes potential bias from inconsistent EF assessment methods. A longer median follow-up period of 3.6 years allowed for an in-depth evaluation of hospitalization, mortality, and myocardial recovery outcomes.

The study is one of the first to specifically evaluate BNP's predictive role of EF recovery and mortality following device implantation and employed well-established clinical prognostic parameters to assess the impact of EF recovery on clinical outcomes in a non-tertiary hospital setting. Notably, this study was conducted at a community-based hospital serving an underserved population, where positive clinical outcomes were observed despite challenges related to unmeasured factors such as medication adherence and socioeconomic status. In 2023, Waterbury, CT, where the hospital is located, had a poverty rate of 23.9%, nearly double the national average of 12.4%. This highlights the significance and real-world applicability of these study’s observations.

Limitations

The study included only patients who underwent primary prevention therapy with ICD/CRT devices, and therefore, the findings cannot be generalized to secondary prevention populations or those with other device types. A small sample size limits the statistical power and generalizability of the results. Additionally, the study was conducted in a single, non-tertiary hospital, which may affect the applicability of the findings to other settings. The retrospective design of the study presents inherent biases related to the inability to control for confounding variables that could affect the outcomes. By excluding patients with early post-device EF assessments, we did not capture the ‘super-responders’, those who experienced an EF recovery within the first few months post-implantation, potentially leading to an underestimation of the prevalence of HFimpEF in this population and indicating the benefit of further studies.

## Conclusions

This study highlights the favorable clinical outcomes associated with HFimpEF compared to persistent HFrEF following device implantation, including a significant reduction in hospitalizations. We observed a 50% decrease in mortality risk in patients with HFimpEF, although this finding did not reach statistical significance. BNP levels emerged as a key predictor of both myocardial recovery and mortality, underscoring its utility in routine clinical practice. Despite these benefits, challenges in HF failure management persist, necessitating individualized follow-up strategies. Future prospective studies are needed to validate these findings and refine risk-stratification strategies for this growing patient population.
